# Efficacy and mechanisms of neuromodulation in the treatment of irritable bowel syndrome

**DOI:** 10.1186/s42234-025-00186-5

**Published:** 2025-09-30

**Authors:** Kaijie Wang, Md Jahangir Alam, Xinya Lan, Fei Li, Jiande D. Z. Chen

**Affiliations:** 1 Division of Gastroenterology and Hepatology, Department of Internal Medicine, University of Michigan, 1150 E Medical Center Dr, Ann Arbor, MI 48109 USA; 2 Department of Gastroenterology, The First Affiliated Hospital of Zhejiang Chinese Medical University (Zhejiang Provincial Hospital of Traditional Chinese Medicine), Hangzhou, Zhejiang 310003 China; 3 Rehabilitation Medicine Center, Fuzhou University Affiliated Provincial Hospital, Fuzhou, China; 4 Shengli Clinical Medical College of Fujian Medical University, Fuzhou, China

**Keywords:** Irritable bowel syndrome, Neuromodulation, Sacral nerve stimulation, Spinal cord stimulation, Transcutaneous auricular vagal nerve stimulation, Percutaneous electrical nerve field stimulation, Transcutaneous electrical acustimulation

## Abstract

Disorders of gut-brain interaction (DGBI), including irritable bowel syndrome (IBS), have a significant impact on patients, reducing their quality of life and work efficiency. Pharmacological therapy is primarily used as a frontline treatment option for treating IBS. However, owing to the heterogeneous characteristics of IBS and its limited pathophysiological understanding, pharmacological therapy is rather disappointing. Therefore, patients with IBS often use alternative therapies, such as electrical neuromodulation, to treat IBS-related symptoms. Neuromodulation includes invasive and noninvasive methods via implanted electrodes and transcutaneous electrodes, respectively. In this manuscript, we reviewed the therapeutic effects of several electrical neuromodulation approaches, including sacral nerve stimulation, spinal cord stimulation, auricular vagal nerve stimulation, and transcutaneous electrical acustimulation, on the symptoms of IBS. Additionally, we discussed the potential mechanisms, adverse effects, advantages, and disadvantages of different neuromodulation treatment methods.

## Introduction

Irritable bowel syndrome (IBS) is a chronic disorder of gut-brain interaction (DGBI) characterized by altered bowel function and abdominal pain (Mearin et al. 2016). Based on the latest Rome IV diagnostic criteria, the prevalence of IBS is about 4% (Sperber et al. 2021), but the actual number of affected individuals is likely much higher. IBS is predominantly found in women and young people (Camilleri 2021). According to the Rome IV criteria, IBS can be categorized into four subtypes, namely, diarrhea-dominant IBS (IBS-D), constipation-dominant irritable bowel syndrome (IBS-C), IBS with a mixed bowel pattern (IBS-M), and IBS unclassified (IBS-U) (Vasant et al. 2021). IBS not only compromises quality of life but also imposes a significant economic burden and may lead to anxiety and depression. The pathophysiology of IBS is complex, including altered intestinal permeability, dysmotility, visceral hypersensitivity, and abnormal bacterial colonization or microbial dysbiosis (Kassinen et al. 2007; Ceuleers et al. 2016; Black et al. 2020). At present, in clinical practice, the treatment of IBS is mainly to relieve symptoms, and the primary drugs used include laxatives (chloride channel activators and guanylate cyclase activators), antidiarrhea agents (like Loperamide and Rifaximin), antispasmodics(like Pinaverium), central neuromodulators (like tricyclic antidepressive agents and elective serotonin reuptake inhibitor) and microecological preparation (like probiotics) (Chey et al. 2015). Gut-directed hypnosis and cognitive behavioral therapy are also recommended to treat overall IBS symptoms (Wang et al. 2024). Nonetheless, the use of antidiarrheals and antispasmodics may paradoxically induce constipation, complicating treatment for patients with severe or varied symptoms. Thus, patients with more complex symptoms of IBS do not respond well to medical therapy. Moreover, significant adverse drug reactions can occur, making alternative or supplemental therapies necessary for many patients.

The digestive system receives innervation from sympathetic and parasympathetic nerves, which interact with sphincters and intramural blood vessels to regulate muscle movement, intestinal hormone release, immune cell activity, and inflammation (Payne et al. 2019). The vagus and sacral nerve (S2-S4) are essential components of the parasympathetic system. Neuromodulation is “a technology impacting on the neural interface.” It refers to the regulation of central, peripheral, or autonomic nervous system activity using electrical, chemical, or magnetic methods (Krames et al. 2009). Compared with surgical treatment, neuromodulation has the advantages of being non-destructive and adjustable. Currently, neuromodulation is mainly used to treat chronic pain, movement disorders, mental disorders and other diseases. Regarding the treatment of GI disorders with neuromodulation, it was first used to treat gastroparesis and achieved effective results (Forster et al. 2001). Subsequently, neuromodulation was gradually applied to treat digestive tract diseases such as obesity (Cigaina 2002), dysmotility disorders and colitis (Bonaz et al. 2016).

Several reviews on neuromodulation for DGBI (Dz Chen et al. 2022), gastrointestinal motility disorders (Yin and Chen 2023; Yin 2025), visceral pain (Alam and Chen 2023) and inflammatory bowel diseases (Yasmin et al. 2022; Pikov 2023) have been published, but there is no review on the efficacy and mechanism of neuromodulation in the treatment of IBS.

The aim of this systematic review was to review the effectiveness of current electrical neuromodulation techniques for IBS, explore their mechanisms, and discuss the potential side effects, benefits, and cost-effectiveness of these approaches.

## Search methods for the review

We searched for relevant literature on PubMed, Web of Science, and Google Scholar from 2000 to 2024, and included only studies published in English. Regarding the treatment of IBS symptoms with various neuromodulation methods, we only included clinical studies. For the mechanisms of neuromodulation treatment of IBS, we also included animal studies. The included clinical studies comprised randomized controlled trials (RCTs), pilot studies, descriptive follow-up studies, and case–control studies. We excluded non-empirical studies, such as editorial letters, conference proceedings, meeting abstracts, commentary, or authors' replies.

For sacral nerve stimulation, we used the following key words to search the literature: ((“sacral nerve stimulation”) OR (sacral neuromodulation) OR (SNS)) AND ((“irritable bowel syndrome”) OR (IBS)).

For spinal cord stimulation, we used the following key words to search the literature: ((“spinal cord stimulation”) OR (SCS)) AND ((“irritable bowel syndrome”) OR (IBS)).

For auricular vagal nerve stimulation, we used the following key words to search the literature: ((“transcutaneous auricular vagal nerve stimulation”) OR (“auricular vagal nerve stimulation”) OR (“noninvasive auricular vagal nerve stimulation”) OR (taVNS) OR (“percutaneous auricular vagal nerve stimulation”) OR (paVNS) OR (“percutaneous electrical nerve field stimulation”) OR (PENFS)) AND ((“irritable bowel syndrome”) OR (IBS)).

For transcutaneous electrical acustimulation, we used the following key words to search the literature: ((“transcutaneous electrical acustimulation”) OR (“transcutaneous electrical stimulation”) OR (TEA)) AND ((“irritable bowel syndrome”) OR (IBS)).

**Fig. 1:**
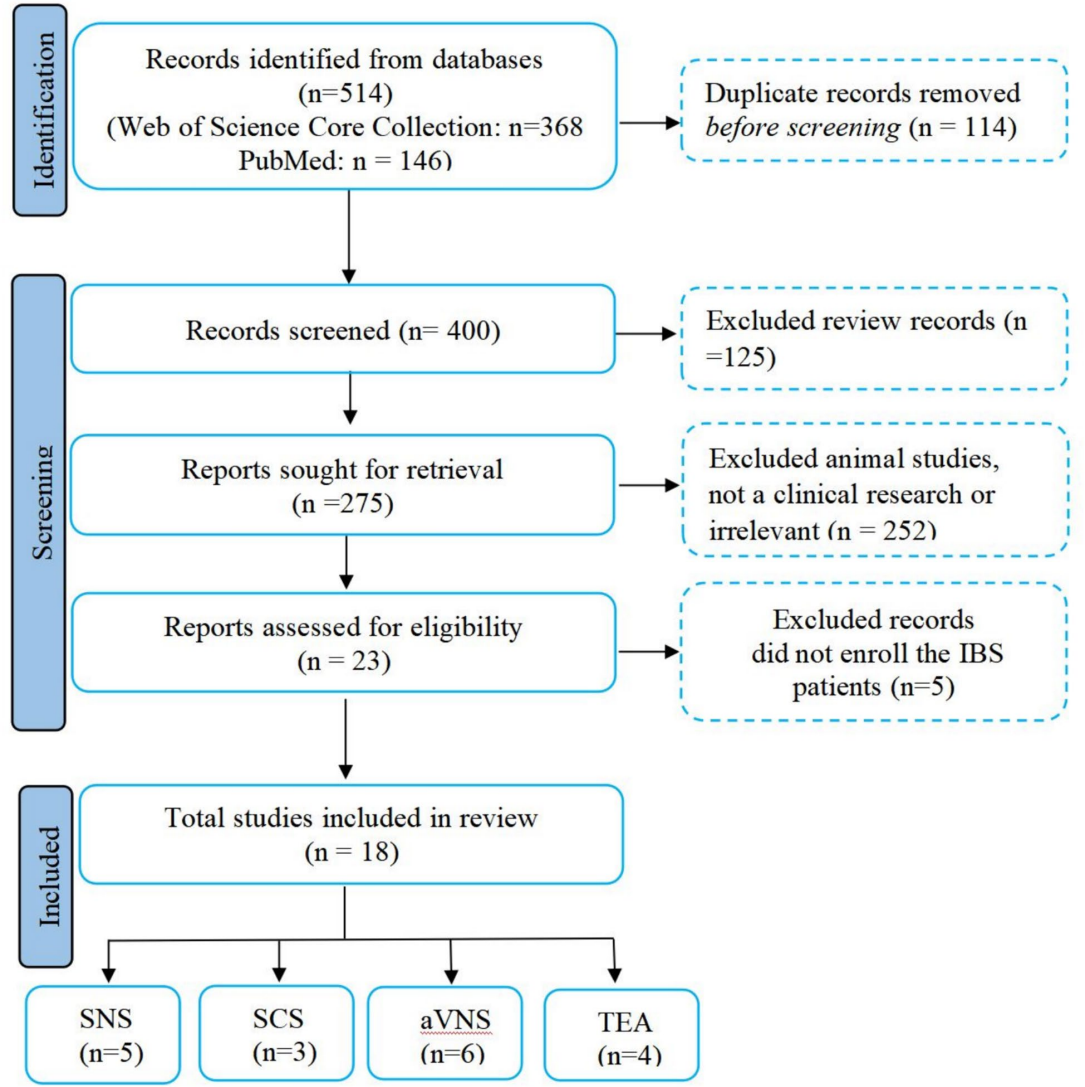
PRISMA flow diagram of study selection. SNS, sacral nerve stimulation; SCS: spinal cord stimulation; aVNS, auricular vagal nerve stimulation (including percutaneous electrical nerve field stimulation, PENFS); TEA, transcutaneous electrical acustimulation

The PRISMA flow diagram of study selection is shown in Fig. 1.

## Literature quality evaluation and bias risk assessment

The Cochrane Risk of Bias V2.0 provided by the Cochrane Collaboration was used to assess the bias risk of the included studies (Sterne et al. 2019), which included five domains (D1-D5, D1: Randomization process, D2: Deviations from intended intervention, D3: Missing outcome data, D4: Measurement of the outcome, D5: Selection of the reported result) Each domain was evaluated as “low risk”, “some concern of risk”, or “high risk”. By combining the results of the five domains, an overall bias can be determined.

## Invasive neuromodulation

### Sacral nerve stimulation

Sacral nerve stimulation (SNS) is a minimally invasive treatment method approved by the Food and Drug Administration (FDA) for overactive bladder (Matzel et al. 1995). This method includes the implantation of an electrode lead in the sacral foramina (S2-S4) and placement of an implantable pulse generator (IPG). Although SNS was initially used for treating urinary incontinence (Stewart et al. 2003), it has also been approved for the treatment of fecal incontinence (Katuwal and Bhullar 2021), and applied for chronic pelvic pain (Martellucci et al. 2012) and chronic idiopathic anal pain (Falletto et al. 2009), as well as other gastrointestinal diseases, such as constipation and IBS. In 2008, Lundby et al. conducted a pilot study for treating IBS symptoms in IBS-D patients in which they used a temporary SNS stimulator for 3 weeks of stimulation (Lundby et al. 2008). They found that the IBS-symptom score (IBS-SSS) was decreased from 48.9 to 28.3 (*P* = 0.004), and the IBS-quality of life (IBS-QoL) score decreased from 99.3 to 59.6 (*P* = 0.009). In 2014, Fassov et al. evaluated the effects of SNS on rectal sensitivity and biomechanical properties in 20 IBS patients (IBS-D and IBS-M) and found that SNS relaxes the rectal wall, making it more sensitive to stretch and less sensitive to cold (Fassov et al. 2014a). Reduced stiffness of the intestinal wall and increased sensitivity to stretching were reported to be associated with improved symptoms in IBS patients. The treatment period of SNS stimulation in this study was a two-month crossover (one-month on and the other month off). SNS was found to significantly reduce IBS-SSS and improve the IBS-QoL scores. At follow-up after 1 year of treatment, the median IBS-SSS was significantly lower than the baseline (Fassov et al. 2014b). This study provided evidence that SNS could reduce IBS symptoms and improve QoL in patients with IBS. They followed the same IBS patients three years later and found that the IBS-SSS score remained significantly lower than the baseline (Fassov et al. 2017). The median IBS-QoL also improved significantly compared with baseline. In 2019, the same group conducted a single-center, double-blind, randomized, placebo-controlled trial that reached similar conclusions, further confirming the efficacy of SNS for treating symptoms in patients with IBS (Fassov et al. 2019). Notably, this study found a significant reduction in abdominal pain (*P* = 0.02) and the number of daily bowel movements (*P* = 0.04) in IBS patients during SNS stimulation. By investigating the same group of patients with multimodal impedance planimetry, it was found that SNS did not play a positive role in treating diarrhea-type and mixed IBS patients through modulation of the postprandial response (Fassov et al. 2020). The clinical studies related to SNS are summarized in Table 1.

**Table 1:** Sacral nerve stimulation for the treatment of IBS symptoms

Articles	Study design	IBS-type	Stimulation mode and parameter	Number of patients	Study quality	Main findings
Lundby L et al. (2008)	Uncontrolled	IBS-D	Temporary	6 (5 females, 1 male)	Not applicable	The irritable bowel syndrome symptom score decreased from 48.9 to 28.3 (P = 0.004). And the irritable bowel syndrome quality of life score decreased from 99.3 to 59.6(P = 0.009)
Fassov J et al. (2014a)	RCT crossover	IBS-D and IBS-M	Turned ON or OFF for the first one month and then to the opposite setting for the next month. Individual parameter settings	20 (15 females, 5 males)	low risk	SNS for diarrhea-predominant and mixed IBS relaxes the rectal wall, while making it more sensitive to stretch and less sensitive to cold. Reduced wall stiffness and increased sensitivity to stretch are associated with improved GSRS-IBS symptom score Sacral nerve stimulation significantly reduces symptoms and improves quality of life of highly selected patients with IBS
Fassov J et al. (2017)	Descriptive follow-up	IBS-D and IBS-M		20 (15 females, 5 males)	Not applicable	SNS continues to be an effective treatment for highly selected patients with diarrhea-predominant or mixed IBS
Fassov J et al. (2019)	RCT Double-blinded, placebo‐controlled crossover	IBS-D and IBS-M	6-week period of SNS (First 4 weeks, subsensory or OFF for 2 weeks and then the opposite for another 2 weeks; suprasensory in the remaining 2 weeks)	21 (15 females, 6 males)	low risk	The IBS-specific symptom score was reduced with borderline significance during stimulation. Pain was significantly reduced during stimulation along with the number of daily bowel movements. Sacral nerve modulation for IBS seems promising
Fassov J et al. (2020)	RCT Double-blinded, placebo‐controlled crossover	IBS-D and IBS-M	week period of SNS (First 4 weeks, subsensory or OFF for 2 weeks and then the opposite for another 2 weeks; suprasensory in the remaining 2 weeks)	20 (14 females, 6 males)	Not applicable	Sacral nerve modulation does not exert its positive treatments effects in diarrhea-predominant and mixed irritable bowel syndrome through a modulation of the postprandial response

*GSRS-IBS* The Gastrointestinal Symptom Rating Scale – Irritable Bowel Syndrome version

As an invasive neuromodulation, SNS also deserves special attention for its possible adverse events. In the 20 patients RCT study conducted by Fassov J et al. (Fassov et al. 2014b), a total of seven patients reported 10 device-related adverse events. Four were classified as mild, one moderate, and five severe (3 patients experienced persistent postoperative pain at the implantation site necessitating relocation of the stimulator, and 2 patients had an elective revision of the lead for suspected migration). Only two device-related adverse events were reported between 1 and 3 years of follow-up in the same cohort (Fassov et al. 2017). Included a case of recurrent migraine resolving with the stimulator turned off for a while and a case of a recurrent tingling sensation under the foot on the side of implantation. The researchers classified these two adverse events as moderate. No life-threatening adverse events have been reported in the current literature. A review focusing on the adverse events of SNS for fecal incontinence found that the most common issues were lack or loss of benefit (48.9%), pain or dysesthesia (27.8%) and complication at the generator implantation site (8.7%) (Bielefeldt 2016).

### Spinal cord stimulation

Electrical stimulation of the dorsal column of the spinal cord has been used to treat severe chronic pain and ischemic pain for a long time. Spinal cord stimulation (SCS) is a technique involving electrical stimulation of specific regions of the spinal cord, particularly targeting the spinal segments corresponding to paresthesia covering the painful areas in patients. It uses an IPG to deliver low-energy electrical currents to the spinal cord, essentially modulating pain signals from that part of the body via an electric stimulation. The first use of SCS for relieving IBS-related symptoms in Humans was reported in 2004 (Krames and Mousad 2004). Krames E et al. reported a case of a patient with IBS treated with SCS who had severe intractable abdominal pain and diarrhea for 30 years and had little response to conservative treatment. A Pisces quadrupolar spinal cord stimulating electrode array was placed percutaneously and advanced to the patient’s T8 vertebra spinal segment. During the first 6 months of SCS, the patient's abdominal pain and diarrhea symptoms were significantly improved. However, 10 months later, the patient started to experience increased pain but remained diarrhea-free. Transverse tripolar stimulation was used in another case report (Rana and Knezevic 2013). The hypothesis was that the maximum dorsal column stimulation with theoretically improved analgesia could be achieved using the multi-lead technology. The patient underwent implantation of a percutaneous permanent lead at the inferior aspect of the T8 level in a tripolar configuration. The patient reported pain relief after SCS was initiated. During the one-year follow-up period, the Maximum pain score was dropped from between 8 and 10 at baseline to 3/10. Meanwhile, the IBS symptom score was dropped from 410 to 180 after one-year SCS. The quality of life and the ability of the patient to function at work were also improved. These two studies (Krames and Mousad 2004; Rana and Knezevic 2013) were single-case reports involving only one subject and were therefore not suitable for quality evaluation.

In 2015, a team from Sweden conducted a randomized crossover SCS trial using on-and-off periods in 10 patients with IBS (Lind et al. 2015). They used a quadripolar SCS lead with electrodes placed around the T5-T8 levels. After electrode implantation, 10 patients were randomly assigned to two groups. One group of patients received stimulation for six weeks and stimulation was Turned off for the next six weeks. The other group of patients received the treatment in a reversed sequence. All patients received stimulation for 12 weeks starting from week 14 (the first 2 weeks of the experiment were the adaptation period). In the 9 patients who completed the study, the median pain scores were significantly reduced from 7 out of 10 to 3 (early stimulation) and 4 (late stimulation). Diarrhea decreased in several patients. This was a randomized controlled trial that described the methods of randomization and blinding during the trial. Overall bias was evaluated as “low risk”. This pilot clinical study provided evidence that SCS might be a viable minimally invasive treatment option for pain management in patients with IBS.

## Noninvasive neuromodulation

### Auricular vagal nerve stimulation

Composed of afferent (80%) and efferent (20%) nerve fibers, the vagus nerve includes fibers projecting from visceral organs to the lower brainstem and fibers connecting the lower brainstem to the viscera. SNS and SCS, described previously, are exciting and potentially effective treatments for severe and refractory IBS Symptoms but are not an acceptable option for most patients due to their invasive nature. Consequently, peripheral, noninvasive neuromodulation techniques are considered for treating IBS. The parasympathetic nervous system is the main component of the autonomic nervous system. It originates from the central nervous system and plays an essential role in controlling and regulating the gastrointestinal tract. Vagus nerve stimulation (VNS) has attracted much attention as a potential neuromodulation method. In 1997, the FDA approved the first implantable VNS device for the treatment of refractory epilepsy (Goggins et al. 2022). So far, the FDA has approved the use of VNS for depression, migraines, and other diseases (George et al. 2007). Traditional VNS, which stimulates the left cervical vagus nerve through a surgically implanted pulse generator device, poses a challenge for many patients due to its invasive nature. Researchers have been developing various neuromodulation methods that stimulate the vagus nerve noninvasively.

The external ear uniquely houses a peripheral branch of the vagus nerve (Peuker and Filler 2002). The anatomical principle of aVNS is that it stimulates the auricular branch of the vagus nerve (ABVN), also known as the Arnold’s nerve (Nomura and Mizuno 1984). The cymba concha is innervated exclusively by the auricular vagal afferent nerve. Therefore, noninvasive stimulation of the auricular vagal afferent nerve has been used as an alternative to invasive VNS (Guo and Gharibani 2023). Two devices used for transcutaneous auricular vagal nerve stimulation (taVNS) and percutaneous auricular vagal nerve stimulation (paVNS, also recognized as percutaneous electrical nerve field stimulation, PENFS) have received FDA-clearance for the treatment of abdominal pain adult and adolescent patients with IBS.

taVNS has been applied to mainly treat neurological disorders such as epilepsy and depression (Ventureyra 2000). So far, there are few published clinical studies on the use of taVNS for treating IBS. In 2020, Mion et al. conducted a trial study involving 12 female patients with IBS who received taVNS treatment for 6 months. Among the nine patients who completed the trial, a significant improvement in symptoms was observed at both 3 and 6 months; however, taVNS did not modify any of the measured variables (Mion et al. 2020). Shi et al. conducted a randomized controlled trial exploring the therapeutic effect of taVNS on abdominal pain and constipation in IBS-C patients and showed that taVNS effectively alleviated both pain and constipation (Shi et al. 2021). Additionally, the study reported improvements in rectal sensitivity and symptoms of anxiety and depression. Another recent clinical study published in 2024 confirmed that taVNS effectively alleviated constipation and abdominal pain in patients with IBS-C, and the investigators suggested that the symptom improvement might be attributed to the integrated effects of taVNS on rectal function (Liu et al. 2024).

There are several clinical studies of PENFS for the treatment of IBS in adolescents. Krasaelap A et al. conducted a randomized, sham-controlled trial involving a total of 51 children with IBS (Krasaelap et al. 2020). After three weeks of stimulation, 30% or more reductions in worst abdominal pain were observed in 59% of patients who received PENFS vs 26% of patients who received the sham stimulation (*P* = 0.024). The patients who received PENFS had a composite pain median score of 7.5 vs 14.4 for the sham group (*P* = 0.026). No significant adverse effects were reported. This study confirmed that PENFS was a safe and effective treatment option for adolescents with IBS. However, this study did not assess the long-term efficacy of the PENFS stimulation. The effects of PENFS on pain were not sustained at follow-up 8–12 weeks after the end of treatment. Another recently published clinical study (Castillo et al. 2023) reported PENFS in 27 IBS patients aged 11 to 18 Years; among 17 patients who completed the 4-week treatment four weeks, PENFS was noted to affect the microbial metabolic pathways: a potential decrease in metabolic pathways related to carbohydrate degradation and long-chain fatty acid (LCFA) biosynthesis. A sustained reduction in abdominal pain, functional disability, and pain catastrophizing persisted at the 2–3 months of follow-up in patients treated with PENFS. A pilot study conducted in 2021 (Bora et al. 2023) investigated the effect of the PENFS therapy on microbiota composition in adolescent IBS patient samples and found no significant alterations in ɑ or β diversity. However, a relatively abundant of *Blautia* species was found among the “excellent responders” (patients with excellent therapeutic response). IBS-SSS (IBS Severity Scoring System), VSI (Visceral Sensitivity Index), and FDI (Functional Disability Inventory) scores were decreased significantly after the PENFS therapy. The clinical studies related to taVNS and PENFS are summarized in Table 2.

**Table 2:** Auricular vagal nerve stimulation for the treatment of IBS symptoms

Articles	Study design	IBS-type	Stimulation mode and parameter	Number of patients	Study quality	Main findings
Mion et al. (2020)	Uncontrolled study, open-label	Not mentioned	TaVNS; pulse width of 250 μs, frequency of 30 Hz. 3 h per day, 5 days a week for 6 months	9 females	Not applicable	There was a significant improvement of symptoms at 3 and 6 months although none of the measured variables were modified by taVNS
Shi X et al. (2021)	Randomized controlled trial	IBS-C	TaVNS; 2s-on, 3s-off, 0.5ms, 25Hz, 0-2mA. Twice a day for 30 min each time, lasting for 4 weeks	42 adults (32 females, 10 males)	low risk	Noninvasive taVNS improves both constipation and abdominal pain in patients with IBS-C
Liu J et al. (2024)	Randomized, controlled, and single-blind	IBS-C	TaVNS; 2s-on, 3s-off, 0.5ms, 25Hz, 0-2mA. once daily (30 min at 3 PM) for 4 weeks	40 adults (30 females, 10 males)	low risk	Noninvasive taVNS effectively improved constipation and abdominal pain symptoms in patients with IBS-C. The alleviation of IBS-C symptoms may be attributed to the integrative effects of taVNS on rectal functions, mediated through vagal, cholinergic, and multiomics mechanisms
Krasaelap A et al. (2020)	Randomized, double-blind Trial	IBS-C, IBS-D, IBS-M	PENFS; 3.2 V, 1 ms pulses of 1 and 10 Hz every 2 s, 2 h on and 2 h off for 120 h. 5 days/week for 4 weeks	50 adolescents (45 females, 5 males)	low risk	Auricular neurostimulation reduced abdominal pain scores and improved well-being in adolescents with irritable bowel syndrome
Castillo DF et al. (2023)	Case–control study	Not mentioned	PENFS; 1 ms pulses of 1 and 10 Hz every 2 s. 5 days/week for 4 weeks	27 adolescents (22 females, 5 males)	high risk	PENFS showed improvements in pain, disability, and catastrophizing. Carbohydrate degradation and LCFA synthesis pathways decreased post-treatment and at follow-up
Bora G et al. (2023)	Uncontrolled study, open-label	IBS-C, IBS-D, IBS-M	PENFS; 3.2 V, 5 days/week for 4 weeks	20 adolescent females	Not applicable	No substantial microbial diversity alterations with PENFS. Excellent therapeutic respondors showed an enrichment of relative abundance of *Blautia*

*LCFA* Long chain fatty acid

Three most common side effects of taVNS included local skin irritation from electrode placement, headache, and nasopharyngitis (Redgrave et al. 2018); other side effects included ear discomfort, adhesive allergy, and syncope due to needle phobia. No serious adverse events were reported.

### Transcutaneous electrical acustimulation

Acupuncture has been utilized for millennia to treat various ailments. A systematic review highlighted that Chinese patients with IBS experienced more substantial benefits from acupuncture compared to pharmacological treatments for IBS (Manheimer et al. 2012). Hegu (LI4), Neiguan (PC6), and Zusanli (ST36) are commonly used acupoints for the treatment of digestive diseases with acupuncture. Transcutaneous electrical acustimulation (TEA), also known as transcutaneous electrical acupoint stimulation (TEAS), offers a noninvasive, simple, cost-effective, and repeatable alternative to traditional acupuncture (Han et al. 1991). TEA is an entirely noninvasive neuromodulation method in which skin electrodes are placed at specific acupuncture points, and the stimulation points are generally in the vicinity of peripheral nerves. TEA is needleless, simple to use, and can be self-administered by patients at home daily or even a few times daily. In a 2004 pilot study (Xing et al. 2004), TEA at ST36 and P6 significantly increased thresholds for rectal sensation, desire to defecate, and pain in IBS patients. However, TEA did not affect rectal tone and compliance. Another pilot study from China in 2004 (Xiao and Liu 2004) confirmed that TEA at acupoints (LI 4, ST 36, UB 57) improved rectal sensory thresholds and related symptoms in IBS-D patients (n = 44). Hu et al. (Hu et al. 2022) conducted an RCT study in 42 IBS-D patients (36 completed the study) with TEA through Hegu (LI4) and Zusanli (ST36) acupoints for one hour, twice a day for one month. TEA was found to significantly improve quality of life (before: 78.55 ± 9.62, after: 85.97 ± 9.49, *P* < 0.0001). Both TEA and sham-TEA reduced abdominal pain; however, TEA was more potent than sham-TEA (*P* = 0.014). Published in 2022, a placebo-controlled randomized clinical trial (Huang et al. 2022) explored the role of TEA in treating abdominal pain and constipation in patients with IBS-C. Fifty-two patients were randomized into two groups: daily TEA for 4 weeks and daily sham-TEA for 4 weeks. TEA was found to improve constipation and abdominal pain. In addition, there was a significant improvement in the quality of life of in these patients. Physiologically, TEA improved colon transit and increased the threshold of rectal sensation and vagal activity. The results of this clinical study revealed the ability of TEA to treat both abdominal pain and constipation in IBS-C patients. The clinical studies related to TEA and their main findings are summarized in Table 3.

**Table 3:** Transcutaneous electrical acustimulation for the treatment of IBS symptoms

Articles	Study design	IBS-type	Stimulation acupoints and parameter	Number of patients	Study quality	Main findings
Xing J et al. (2004)	Uncontrolled study	IBS-D	ST36, P6	7 adults	high risk	TEA can reduce rectal sensitivity in IBS patients. The effect is not modulated by changes in rectal biomechanics
Xiao WB et al. (2004)	Uncontrolled study	IBS-D, IBS-C	LI4, ST36, UB57; 0.3 ms, 100 Hz, 26-30 mA. 30 min, two times per week for 2 months	44 adults (22 females, 22 males)	high risk	Acupoint TENS improved rectal sensory thresholds and related symptoms in IBS-D patients
Hu P et al. (2022)	Randomized controlled trial	IBS-D	ST36: 2 s-on, 3 s-off, 25 Hz, 0.5 ms, current intensity for patients. L14: 0.1 s-on, 0.4 s-off, 100 Hz, 0.5 ms, current intensity for patients. 30 min, twice per day, one month	36 adults (19 females, 17 males)	low risk	TEA at LI4 and ST36 improves abdominal pain and quality of life of patients with IBS-D
Huang Z et al. (2022)	Placebo-controlled randomized clinical trial	IBS-C	PC6, ST36; 2 s-on, 3 s-off, 25 Hz, 0.5 ms, maximum level tolerated (2-10 mA). 1 h, twice daily, lasting for 4 weeks	52 adults (18 females, 34 males)	low risk	TEA improves constipation and symptoms of IBS by accelerating colon transit and reducing rectal sensation

### Mechanisms

Mechanisms underlying neuromodulation treatments for IBS are intricately linked to the disorder’s complex pathophysiology, involving central, peripheral, and psychological factors. It is generally accepted that stress, as well as early-life stress, contributes to the development and progression of IBS, a notion that has been confirmed in animal studies (Tao et al. 2023). The enteric nervous system (ENS), autonomic nervous system (ANS), and central nervous system (CNS) are partially or collectively altered, which can lead to dysregulation of the brain-gut axis, resulting in IBS. This dysregulation causes visceral hypersensitivity, currently considered a primary cause of IBS (Ceuleers et al. 2016). IBS patients often exhibit low-grade intestinal inflammation, characterized by abnormal immune cells, heightened activity of pro-inflammatory cytokines, eosinophils and mast cells, increased numbers of mucosal immunocytes, and reduced expression of anti-inflammatory cytokines (Bennet et al. 2016; Arzani et al. 2020).

**Fig. 2:**
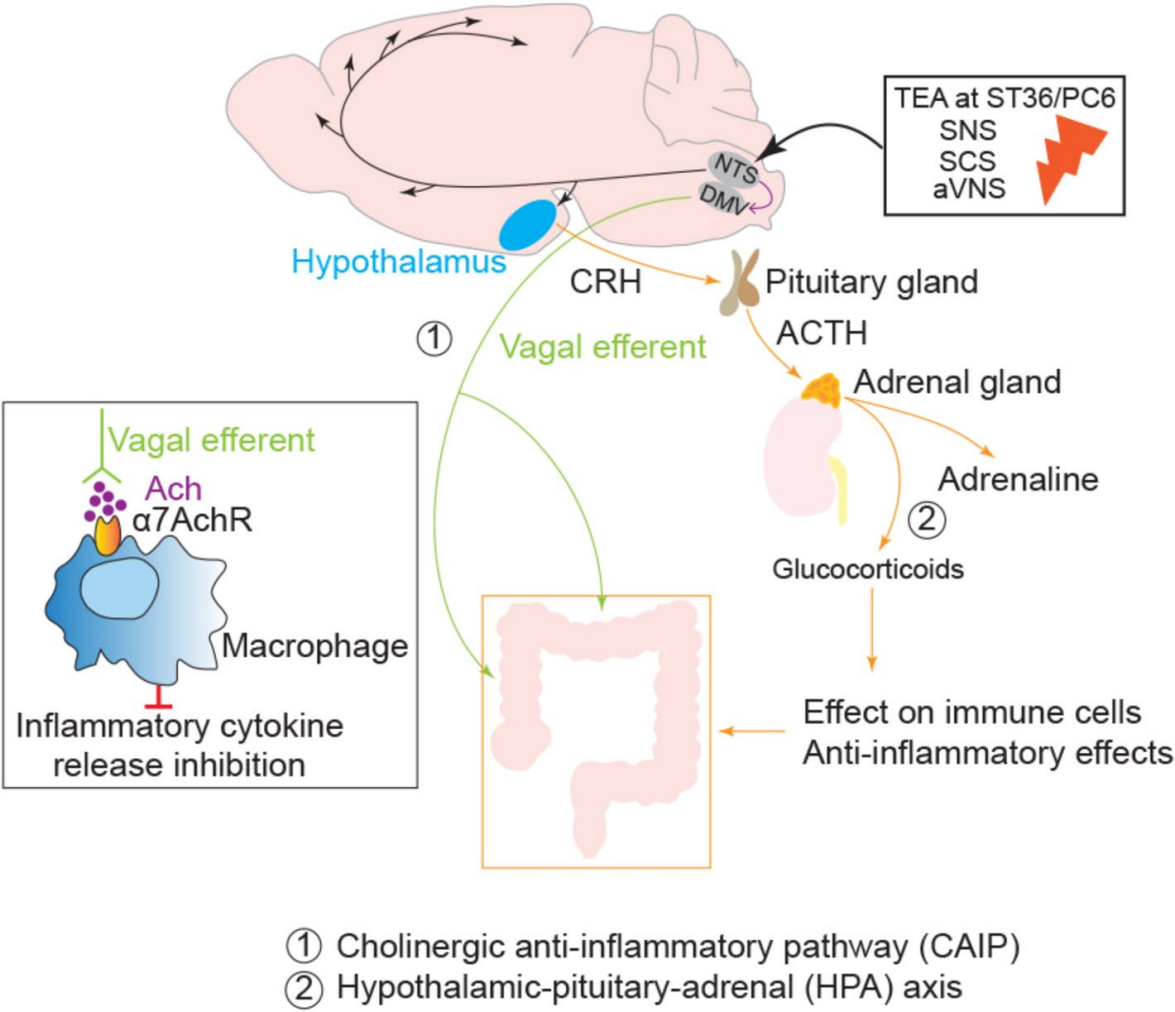
The possible mechanisms underlying neuromodulation treatments for IBS. NTS: nucleus tractus solitarius; DMV: dorsal motor nucleus of the vagus; CRH: corticotrophin-releasing hormone; ACTH: adrenocorticotrophic hormone; ACH: acetylcholine; ɑ7nAChR: ɑ7 nicotinic acetylcholine receptor

Previous studies have suggested the role of the vagus nerve in various neuromodulation methods. IBS patients have dysregulated autonomic nervous systems, primarily evidenced by reduced vagal activity (Sadowski et al. 2020; Cheng et al. 2013). The vagus nerve regulates the gut-brain-microbiome axis bidirectionally and interacts with the immune system. Vagal afferents detect peripheral inflammatory responses and relay signals to the brain stem, which in turn sends efferent signals to modulate immune responses. The specific mechanisms involve pathways such as the anti-inflammatory hypothalamic–pituitary–adrenal (HPA) axis and cholinergic anti-inflammatory pathways (CAIP) (Tracey 2002). Two immune cell types, β_2_ adrenergic receptor positive CD4 + T cells and ɑ7 nicotinic acetylcholine receptor (ɑ7nAChR) expressing macrophages, play a vital role in the CAIP. Under pathological conditions, the vagal afferents are stimulated by peripheral pro-inflammatory cytokines and transmit signals to the nucleus tractus solitarius (NTS) in the CNS, which sends signals to higher neurons. Within the HPA axis, after the brain integrates the signal, corticotropin-releasing factors are activated, leading to glucocorticoid release from the adrenal glands, known for their anti-inflammatory effects, thus reducing systemic inflammation. In the CAIP, the vagus efferent nerve releases acetylcholine (ACh) onto ɑ7nAChR expressed in intestinal and splenic macrophages, inhibiting the release of pro-inflammatory cytokines, thereby improving the anti-inflammatory effect (Meregnani et al. 2011). Studies have demonstrated an inverse correlation between vagal tone and plasma epinephrine levels in IBS patients (Pellissier et al. 2014). The number of colonic 5-HT-positive cells in the gut May be related to abdominal pain. 5-HT is a classic pain-related substance that is widely present in Mammalian tissues. Compared with Healthy controls, the release of 5-HT in IBS patients is significantly increased and correlated with the degree of abdominal pain (Cremon et al. 2011). The possible mechanisms of action are illustrated in Fig. 2.

The sacral nerves contain both autonomic and somatic efferents regulating the functions of the colon and rectum. The mechanism of SNS is not fully understood, but some studies have shown that afferent nerves play a crucial role in modulating spinal reflexes and brain centers (Wachter et al. 2020). Discovering the effects of the SNS on organs not innervated by the sacral nerves, such as the stomach, also supports this viewpoint and demonstrates a spinal afferent and vagal efferent pathway of SNS (Ye et al. 2020). The mechanism of SNS action can be divided into two pathways. The first involves the direct activation of sacral efferent nerves by SNS, promoting the release of ACh in the colon and diminishing the release of pro-inflammatory cytokines through the CAIP. The other pathway includes the spinal afferent-brainstem-vagal efferent route through the CNS. Previous studies showed that SNS improved 2,4,6-trinitrobenzene sulfonic acid (TNBS)-induced colitis in rats (Tu et al. 2020a), and the effect was noted only in groups with intact afferent sacral and vagus efferent nerves, suggesting that the anti-inflammatory effect on TNBS-induced colitis was mediated via the spinal afferent-brainstem-vagal efferent-colon pathway. Recent animal experimental results aligned with these findings, demonstrating that SNS can alleviate visceral hypersensitivity in rats, likely tied to regulating ANS functions and suppressing excessive mast cell activation in colon tissue (Jin et al. 2021). Additionally, SNS was reported to increase the expression of intestinal tight junction proteins, such as zonula occludens-1, occludin, claudin-1, and junctional adhesion molecule-A in the colon tissue (Tu et al. 2020b).

The mechanism of SCS action is still unclear, but one hypothesis is that SCS inhibits pain pathways in the dorsal column of the spinal cord. Animal experiments showed that SCS normalized visceromotor reflexes (VMR) in a rat model of post-inflammatory colonic hypersensitivity (Greenwood-Van Meerveld et al. 2005). SCS was also reported to activate the anterior pretectal nucleus, potentially enhancing descending pain inhibitory pathways via a supraspinal mechanism (Oakley and Prager 2002). Chronic visceral nociception is transmitted in the central nervous system through the postsynaptic dorsal column (PSDC) pathway and lateral spinothalamic tracts (LSTT), and SCS may ameliorates pain by modulation of the above pathways (Krames and Foreman 2007). Furthermore, the antidromic activation of sensory nerves that innervate the gut plays a significant role in the effectiveness of SCS, as evidenced by various experimental studies (Tanaka et al. 2001).

The auricular vagus nerve projects directly to the NTS, influencing the central and autonomic nervous systems (Butt et al. 2020). Its anti-inflammatory effect of reducing pro-inflammatory cytokines levels can be achieved by CAIP, as described previously. Functional magnetic resonance imaging (fMRI) scans have provided evidence in humans that the central projections of the ABVN are consistent with the "classical" central vagal projections, including widespread activity in the ipsilateral NTS, bilateral spinal trigeminal nucleus, dorsal raphe, locus coeruleus, and contralateral parabrachial area, amygdala, and nucleus accumbens, and can be accessed non-invasively via the external ear (Frangos et al. 2015). The NTS is a relay station in the brain, forwarding signals to the rostral ventral medulla, hypothalamus, amygdala, and spinal cord. Findings from a study (Babygirija et al. 2017) using a rat model of post-inflammatory hyperalgesia showed that PENFS reduced the firing of neurons in the central nucleus of the amygdala (CeA) and lumbar spinal cord by approximately 60% and 50%, respectively, thus diminishing visceral hyperalgesia. These findings suggested that PENFS might have dual effects on the visceromotor response and ascending central pathways. The limbic system, which includes the amygdala, plays a role in mediating instinctive and emotional behaviors, including the emotional response to pain, further underscoring the broad influence of PENFS on pain perception. Mechanistically, taVNS was reported to decrease the serum levels of TNF-α and IL-6, as well as plasma levels of 5-HT, while enhancing vagal activity in patients with IBS-C (Shi et al. 2021). Studies indicated that anxiety and depression symptoms, which contribute to the altered central processing of visceral stimuli in IBS patients, could be alleviated by taVNS. This alleviation of psychiatric symptoms might be a mechanism through which taVNS reduces abdominal pain (Elsenbruch et al. 2010). taVNS was also reported to increase rectal sensation in patients with IBS-C, reflected as decreased sensation threshold to rectal distention (Shi et al. 2021). Several clinical studies (Shi et al. 2021; Clancy et al. 2014) have found that taVNS can enhance parasympathetic activity and gastrointestinal motility. Behavioral and electrophysiological studies have found that taVNS selectively affected the GABAergic system in the motor system contralateral to the stimulated ear (Keute et al. 2018). In brief, both PENFS and taVNS activate vagal afferent fibers by stimulating ABVN. These treatments exert therapeutic effects on IBS patients by modulating central brain pathways and activating anti-inflammatory pathways.

Numerous clinical studies have demonstrated the efficacy of acupuncture in treating IBS, attributing its benefits to regulating gastrointestinal motility, visceral hypersensitivity, brain-gut axis, neuroendocrine system, and immune function (Ma et al. 2014). Experiments in rats demonstrated that EA at ST36 improved colon transit, mediated by autonomic mechanisms (Wang et al. 2019). Moreover, EA reversed autonomic dysfunction in rats with Loperamide-induced constipation by increasing parasympathetic activity and decreasing sympathetic activity (Wang et al. 2019). In a rodent model IBS, EA-produced visceral analgesia was found to be associated with the reversal of the enhanced excitability of DRG neurons (Xu et al. 2009). Clinical studies also support the enhancive effect of EA and TEA on parasympathetic activity (Zhang et al. 2019; Chen et al. 2013). Furthermore, EA at ST36 was reported to inhibit pro-inflammatory cytokines (TNF-ɑ, IL-1β, and IL-6) through the autonomic mechanism and improve intestinal inflammation in a rodent model of colitis (Jin et al. 2019). Huang et al. reported increased vagal activity and decreased sympathetic activity with TEA at ST36 in patients with IBS-C (Huang et al. 2022). However, some clinical studies and animal experiments reported that TEA or EA did not change the autonomic function (Hu et al. 2022; He et al. 2013). These inconsistencies might be attributed to variations in experimental conditions, stimulation parameters, acupoint selection, and differences in the populations studied. Taken together, EA/TEA has been shown to suppress intestinal inflammation; generally, the mechanism is thought to be similar to taVNS and potentially facilitated by the vagal-mediated anti-inflammatory pathway. Ma et al. used fMRI to study the correlations between brain functional connection, interaction, segregation, and acupuncture stimulation in IBS (Ma et al. 2020). Their results indicated that the changes in the brain functional connection, interaction, and segregation in the hippocampus, middle and superior occipital gyrus, cerebellum, and lingual gyrus might be related to acupuncture stimulation, and these changes were related to the relief of symptoms in patients. Another clinical study (acupoints of ST25 and ST37) involving 62 IBS-D patients showed that EA significantly reduced the expression of 5-HT, 5-HT3R, and 5-HT4R in the colonic mucosa (Zhao et al. 2015). Animal experiments also found that acupuncture at heterotopic acupoints increased distal colonic motility by activating C-fibers (Axons that conduct at low speeds) of somatic afferent nerves and M3 receptors, suggesting the mechanisms of EA/TEA might alleviate constipation in IBS patients (Gao et al. 2015). A study in dogs also proved that EA at ST36 could restore rectal distension-induced impairment in both colonic contraction and transit by enhancing vagal activity and mediating via the cholinergic pathway (Jin et al. 2015). The exact neural pathways involved in the regulation of the autonomic nervous system are not fully understood. Previous findings seem to support following mechanisms of EA/TEA: 1). EA/TEA activates peripheral nerves, sending afferent signals to the CNS; the CNS after processing the afferent signals sends enhanced parasympathetic efferent signals to the gut, differentially affecting excitatory and inhibitory neurons of the ENS, and thus restoring GI motility (Liang et al. 2018). 2). Enhanced parasympathetic activity leads to the release of acetylcholine, resulting in suppression of gastrointestinal inflammation via the CAIP; 3) suppression of low-grade inflammation leads to reduction of visceral hypersensitivity, resulting in improvement in visceral pain.

## Discussion

We have reviewed the effects of various neuromodulation therapies for IBS and their potential underlying mechanisms. In this section, we discuss the merits and drawbacks, along with the cost-effectiveness of neuromodulation therapies for IBS. As opposed to Rome III, Rome IV discarded the term 'discomfort' in the diagnostic criteria for IBS, focusing solely on 'abdominal pain' as the primary diagnostic symptom. Since abdominal pain is a prevalent symptom and a crucial indicator of the severity in IBS patients (Spiegel et al. 2008) reduction in pain is considered a primary treatment objective.

SNS is known to have a therapeutic effect on fecal incontinence; however, clinical studies so far have found that SNS has a poor therapeutic effect on patients with slow-transit constipation (Altomare et al. 2021), and has no effect on gastrointestinal motility. However, in a few clinical studies of SNS for IBS mentioned in this review, SNS seemed to exert a therapeutic effect on IBS. This enhancive effect might be attributed to the exclusion of IBS-C patients from these studies. The stimulation parameters of SNS for the treatment of IBS are largely similar, which may be explained by the fact that most clinical studies originate from the same research team (Lundby et al. 2008; Fassov et al. 2014a, 2019, 2020). A characteristic feature of their protocol was that during the first four weeks of treatment, patients received either subsensory stimulation or OFF for two weeks, followed by the opposite condition for another two weeks. The physiological similarities between diarrhea in IBS and fecal incontinence suggest a potential basis for the successful application of SNS in treating IBS-D or IBS-M (Thin et al. 2013). SNS is more costly than noninvasive methods. However, according to the modeling results, it is cost-effective in the long-term treatment of IBS (> 7 years) (Tipsmark et al. 2016). In comparison to pharmacotherapy, the safety of neuromodulation devices, especially invasive ones like SNS, emerges as a critical consideration. Although no serious adverse events have been reported in the current literature, permanently implanted devices might become a potential source of tissue irritation or infection beyond its period of utility or the duration of a trial. Hence, continuous monitoring and care of patients are recommended as long as the device remains in the patient body.

For SCS, the specific placement and extent of the electrode lead deserve discussion. Theoretically, multiple electrodes could increase the activation rate in the dorsal column area and activate a wider range of nervous tissue. However, an excessively wide electric field may result in stimulation of the dorsal root and cause reflex muscle contractions. The tripolar device used by Rana MV et al. prevents the electric field from extending beyond the anodes and generates directional energy covering a wider area while ensuring patient safety (Rana and Knezevic 2013). Additionally, it has been shown sustained improvement in abdominal and thoracic spine pain among patients, indicating its potential for long-term relief. SCS is an invasive neuromodulation method; however, most patients chosen to retain the SCS system after clinical trials (Lind et al. 2015). This indicates that SCS might be an effective treatment for some patients with long-standing conditions, particularly those with severe abdominal pain.

Targeting central pain pathways through neuromodulation presents a new strategy for treating DGBI. The significant effect of PENFS on relieving pain symptoms in pediatric patients with IBS underscores its potential as a clinical treatment, particularly for those with abdominal pain as a primary symptom. Cost–benefit and cost-minimization analysis of the clinical studies we mentioned above shows that PENFS for IBS in adolescents provides significant cost savings for patients and insurance companies (Shah et al. 2024). The clinical trial of Krasaelap A et al. did not survey the long-term effects of PENFS (Krasaelap et al. 2020). The results of a prospective cohort study of 20 patients with functional abdominal pain disorders followed for up to 1 Year showed that some effects were sustained at 6–12 months post-treatment (Santucci et al. 2022). Additionally, dysbiosis is a common feature in IBS; clinical studies of PENFS discovered that it might affect the intestinal microbiota (Castillo et al. 2023; Bora et al. 2023), possibly through the CAIP. The disadvantage of the PENFS method is that it uses short needles and the procedure has to be performed by Healthcare provides. In these clinical studies investigating the use of PENFS for adolescent IBS, the stimulation parameters were similar, consisting of 3.2 V with 1 ms pulses at 1 and 10 Hz every 2 s (Krasaelap et al. 2020; Castillo et al. 2023; Bora et al. 2023). The treatment regimen lasted for 4 weeks, with sessions conducted 5 days per week.

A few published RCTs seemed to demonstrate that taVNS and TEA might be good treatment options for IBS. Unlike the method of PENFS, taVNS and TEA can be self-administered since they do not use any needles. The distinctive advantage of taVNS/TEA for IBS-C is that taVNS/TEA improves both pain and constipation, which is uniquely attractive as pharmacological treatment of pain often leads to worsening of constipation. In addition, taVNS/TEA ameliorates major pathophysiologies of IBS-C, such as visceral hypersensitivity and slow colon transit. More studies, especially multi-center studies are needed to further establish their clinical efficacy in treating IBS. Several clinical studies from China investigating TEA and taVNS for the treatment of IBS employed similar stimulation parameters (2 s on, 3 s off, 0.5 ms, 25 Hz, 0.5–5.0 mA) (Shi et al. 2021; Liu et al. 2024; Hu et al. 2022; Huang et al. 2022). This consistency was likely attributable to the use of devices manufactured by the same company, with earlier studies having demonstrated that these parameters could effectively improve gastrointestinal symptoms in IBS patients.

In addition to multi-center pivotal clinical studies, basic animal studies are also needed to explore mechanisms involved in the ameliorating effects of neuromodulation on visceral pain in IBS. Peripherally, neuromodulation may improve visceral hypersensitivity by suppressing low-grade inflammation via the cholinergic anti-inflammatory pathway; it may also improve intestinal barrier function and suppress sensitization of sensory neurons. Centrally, studies are needed to explore possible mechanisms of neuromodulation involving ascending pain transmission pathway and descending pain inhibitory pathway. It is also of interest to investigate the autonomic mechanisms involving both afferent and efferent pathways.

In conclusion, neuromodulation has a great potential for the treatment of IBS. Compared to invasive neuromodulation methods such as SNS and SCS, noninvasive neuromodulation methods (taVNS, PENFS and TEA) hold a potential for future development due to their excellent safety profile and ease of application. Continued clinical and basic research is essential to establish the efficacy further and understand the underlying mechanisms of neuromodulation in treating IBS. Future research will be crucial in optimizing neuromodulation strategies to enhance patient outcomes and broaden the scope of effective treatments available for IBS.

## Data Availability

No datasets were generated or analysed during the current study.
